# Metallothionein1A Regulates Rhizobial Infection and Nodulation in *Phaseolus vulgaris*

**DOI:** 10.3390/ijms23031491

**Published:** 2022-01-27

**Authors:** Citlali Fonseca-García, Claudia Marina López-García, Ronal Pacheco, Elisabeth Armada, Noreide Nava, Rocío Pérez-Aguilar, Jorge Solis-Miranda, Carmen Quinto

**Affiliations:** Departamento de Biología Molecular de Plantas, Instituto de Biotecnología, Universidad Nacional Autónoma de Mexico, Avenida Universidad 2001, Colonia Chamilpa, Cuernavaca 62210, Morelos, Mexico; fonseca-garcia@berkeley.edu (C.F.-G.); marinalopez2508@gmail.com (C.M.L.-G.); ronal.pacheco@ibt.unam.mx (R.P.); eliarm_22@hotmail.com (E.A.); noreide.nava@ibt.unam.mx (N.N.); rociobio2103@gmail.com (R.P.-A.); jorge.solis@ibt.unam.mx (J.S.-M.)

**Keywords:** metallothionein, nodule symbiosis, common bean, rhizobial infection, reactive oxygen species

## Abstract

Metallothioneins (MTs) constitute a heterogeneous family of ubiquitous metal ion-binding proteins. In plants, MTs participate in the regulation of cell growth and proliferation, protection against heavy metal stress, oxidative stress responses, and responses to pathogen attack. Despite their wide variety of functions, the role of MTs in symbiotic associations, specifically nodule-fabacean symbiosis, is poorly understood. Here, we analyzed the role of the *PvMT1A* gene in *Phaseolus vulgaris*-*Rhizobium tropici* symbiosis using bioinformatics and reverse genetics approaches. Using in silico analysis, we identified six genes encoding MTs in *P. vulgaris*, which were clustered into three of the four classes described in plants. *PvMT1A* transcript levels were significantly higher in roots inoculated with *R. tropici* at 7 and 30 days post inoculation (dpi) than in non-inoculated roots. Functional analysis showed that downregulating *PvMT1A* by RNA interference (RNAi) reduced the number of infection events at 7 and 10 dpi and the number of nodules at 14 and 21 dpi. In addition, nodule development was negatively affected in *PvMT1A*:RNAi transgenic roots, and these nodules displayed a reduced nitrogen fixation rate at 21 dpi. These results strongly suggest that *PvMT1A* plays an important role in the infection process and nodule development in *P. vulgaris* during rhizobial symbiosis.

## 1. Introduction

The fabacean common bean (*Phaseolus vulgaris* L.) can establish a symbiotic relationship with soil bacteria known as rhizobia. This symbiotic interaction between fabaceans and rhizobia, hereafter referred to as fabacean-rhizobia symbiosis, begins with an exchange of chemical signals, in which the roots exude flavonoids that induce the synthesis and secretion of lipo-chitooligosaccharides, i.e., nodulation factors (NFs), by the rhizobia [1]. NFs are signaling molecules that are perceived by specific receptors located on the plasma membrane of root hair cells [2], triggering a signaling cascade leading to infection by the rhizobia and cortical cell division [3]. Rhizobial infection normally occurs through root hairs [4], which tips curl in the presence of the rhizobia, forming “infection pockets” [5]. This process is followed by invagination of the plasma membrane, which develops tubular structures called infection threads (ITs). ITs function as tunnels that guide the rhizobia toward the cortical cells, where the nodule primordia form [4,6]. Subsequently, the rhizobia are internalized in the cells of the growing nodules via a process similar to endocytosis and are surrounded by a membrane of plant origin. The bacteria then differentiate into bacteroids and, together with the symbiosomal membrane (a plant-derived membrane), give rise to specialized nitrogen-fixing organelles called symbiosomes [2,7]. The beneficial feature of this symbiosis lies in a niche for the bacteria to live as an endophyte on host costs, thus avoiding the competition with the other soil microbiota, in exchange for the fixed nitrogen (ammonia) provided to the host [8,9].

The mechanisms underlying the establishment of fabacean-rhizobia symbiosis have been widely studied, demonstrating that symbiosis is a complex process involving several molecular, cellular, and physiological mechanisms [4,5,7]. However, many unanswered questions remain, and some of the regulatory mechanisms have yet to be fully elucidated. Comparative transcriptomic analyses of fabacean roots inoculated with rhizobia versus non-inoculated roots have shown that fabacean-rhizobia symbiosis affects the expression of numerous genes, pointing to their participation in this symbiotic process. In a transcriptomic analysis of symbiotic *P. vulgaris* roots previously carried out by our group [10], a gene encoding metallothionein (MT) showed an increased expression in hairy roots in response to rhizobial inoculation. MTs are low molecular weight proteins rich in cysteine motifs. According to the cysteine rearrangement, plant MTs are classified into four types: MT1, MT2, MT3, and MT4 [11]. MT proteins are widely distributed in vascular plants, and their gene expression profiles are tissue specific [12]. For instance, *MT1* genes are mainly expressed in roots, while *MT2* genes are highly expressed in leaves. *MT3* and *MT4* genes are mainly expressed in fruits and seeds, respectively [12,13]. Subcellular localization analyses have shown that MTs are primarily located in the cytoplasm, and they are also found in the nucleus [14,15].

MTs have been shown to be involved in oxidative stress in response to biotic and abiotic factors. The promoter of the metallothionein-like *cgMT1* from the actinorhizal tree *Casuarina glauca* Sieber ex Spreng. is activated by wounding and by H_2_O_2_. Likewise, the strong promoter activity of *cgMT1* was observed in transgenic *Arabidopsis thaliana* (L.) Heynh plants inoculated with the bacterial pathogen *Xanthomonas campestris* pv*. Campestris* [16]. Furthermore, the heterologous expression of *PpMT2* from *Physcomitrella patens* (Hedw.) Bruch and Schimp. reduced the accumulation of H_2_O_2_ and O_2_^−^ in arabidopsis after heavy metal treatment [17]. In rice, cytokinins negatively regulate *OsMT2b*, and analysis of overexpression and knockdown mutants showed that *OsMT2b* regulates seed germination and root development by modulating internal levels of cytokinins [18]. In addition, the overexpression of *GhMT3*, a cotton *MT* gene, increased tolerance to oxidative stress by scavenging reactive oxygen species (ROS) and Zn(II) binding in tobacco plants [19]. Moreover, at low temperatures, *At*MT2A regulates ROS balance under oxidative stress in *A. thaliana* plants [15]. MTs improve tolerance to heavy metals in nodules during fabacean-rhizobia symbiosis. *Rhizobium leguminosarum* bv*. viciae* carrying MTs from pea (*Pisum sativum* L.) developed nodules that were tolerant to toxic concentrations of cadmium (Cd) [20]. In addition, the heterologous expression of arabidopsis *mt4a* in *Medicago truncatula* Gaertn. induced greater tolerance to copper [21]. However, although the roles of *MT* genes in heavy metal tolerance are well known, to our knowledge, no functional studies of *MT* genes in fabacean-nodule symbiosis have been reported.

As mentioned above, our previous transcriptomic analysis of transgenic common bean roots [10] showed that an *MT* gene is induced by rhizobial inoculation at 7 days post inoculation (dpi). This prompted us to investigate the function of this gene in *P. vulgaris*-*R. tropici* symbiosis by real-time quantitative PCR (qPCR) analysis and reverse genetics approaches. 

## 2. Results

### 2.1. MT Genes Constitute a Small Gene Family in P. vulgaris

To analyze the role of *P. vulgaris* MTs in the symbiotic interaction with rhizobia, we first identified the metallothionein genes in the *P. vulgaris* genome by searching the Phytozome database [22] via BLASTP analysis. Six *MT* genes were identified in the genome of *P. vulgaris* (Table S1). The PvMTs were grouped into three classes based on their homology with the MTs from *A. thaliana* and the presence of characteristic motifs and domains: MT1 (Phvul.L001645.1, Phvul.L001745.1, and Phvul.010G009500.1), MT2 (Phvul.008G101800.1), and MT4 (Phvul.008G133400.1 and Phvul.008G133200.1) (Table S2). All PvMT protein sequences showed at least two characteristic cysteine motifs (Figure S1) and the domain IPR000316 (Plant EC metallothionein-like protein), the characteristic domain of the family 15 of MTs in plants. The predicted PvMT proteins have similar theoretical molecular weights ranging from 7.12 to 8.09 kDa and a variable isoelectric point (IP) ranging from 4.33 to 7.86. The PvMT1 and PvMT2 classes showed the most acidic IP values, while the PvMT4 class showed the most basic IP values (Table S3).

In silico mapping of the gene loci revealed that the six *PvMT* genes were distributed only on chromosomes 8 and 10, and all *PvMT1* genes are clustered on chromosome 10 (Figure S2). Gene duplication analysis indicated that tandem, dispersal, and proximal duplications contributed to the expansion of *PvMTs* (Figure S3). In particular, tandem duplication was present in two *PvMT1* genes, Phvul.L001645.1 and Phvul.L001745.1, which share 100% identity and only diverge by 1 nucleotide. Analysis of exon-intron organization showed that the gene structure is highly conserved among *PvMT* genes, with an intron and two exons flanked by the corresponding UTR ends (Figure S4). However, the intron size was variable among *PvMT* classes, where *PvMT1* had the longest intron, followed by *PvMT2* and *PvMT4*.

**Figure 1:**
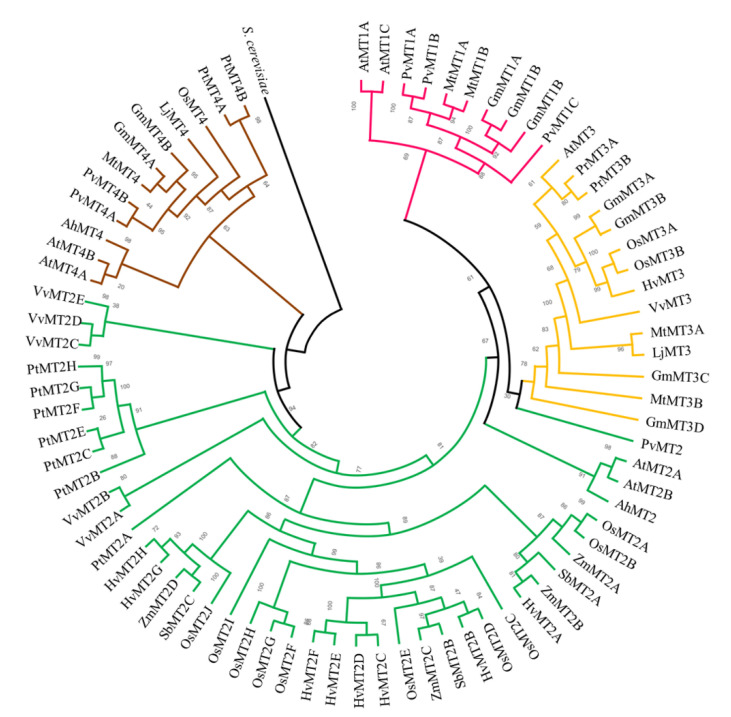
Evolutionary relationships among MTs. Rooted approximately maximum-likelihood phylogenetic tree inferred from 77 MTs identified in 12 plant species: *P. vulgaris* (Ph), *G. max* (Gm), *L. japonicus* (Lj), *M. truncatula* (Mt), *A. thaliana* (At), *V. vinifera* (Vv), *A. hypochondriacus* (Ah), *P. trichocarpa* (Pt), *O. sativa* (Os), *S. bicolor* (Sb), *Z. mays* (Zm), and *H. vulgare* (Hv). The clades are shown in different colors according to the MT classes: MT1, pink; MT2, green; MT3, yellow; MT4, brown. A sequence from *Saccharomyces cerevisiae* was used as the outgroup. The phylogenetic tree was constructed using IQ-TREE software with the Dayhoff substitution model with 1000 bootstrap iterations.

To gain further insight into the phylogeny of PvMT proteins, we reconstructed a maximum-likelihood phylogenetic tree using 77 full-length MT sequences from several species belonging to different groups of plants, fabaceans, nonfabacean dicotyledonous, and monocotyledonous (see Materials and Methods) (Table S1, Figure 1) where *MT* genes have been identified and other important cereal species that have the complete genome on the Phytozome database. In relation to the MT family of *A. thaliana*, the 77 MT protein sequences were phylogenetically grouped into four major clades represented by the ancient classes: MT1, MT2, MT3, and MT4. Interestingly, the MT proteins of fabaceans were mainly distributed in clades MT1, MT3, and MT4, while most monocotyledonous MTs were clustered in MT2.

### 2.2. PvMT1A Expression Changes in Response to R. tropici Inoculation

*MT* genes showed tissue-specific expression patterns in different plant systems [23,24,25], with *MT1* class genes most highly expressed in roots [26]. We previously performed a transcriptomic analysis comparing *P. vulgaris* roots inoculated with *R. tropici* at 7 dpi with non-inoculated roots at the same sampling time [10], which revealed the inoculation-induced upregulation of *PvMT1A* (Phvul.L001645.1). Interestingly, this was the only *MT* gene that was upregulated in *P. vulgaris* following rhizobial inoculation at 7 dpi, suggesting it participates in this stage of the symbiotic process (Figure S5A). In addition, when we compared our results with the PvGEA [27] data, the *PvMT1A* gene was most highly expressed in both non-inoculated roots and inoculated roots with *R. tropici* (Figure S5B). However, as mentioned above, *PvMT1A* presented a tandem duplication of the *PvMT1B* gene Phvul.L001745.1, sharing 100% identity, and since it was not possible to discriminate between the two sequences, we assumed that the observed expression profile is shared by both genes.

**Figure 2:**
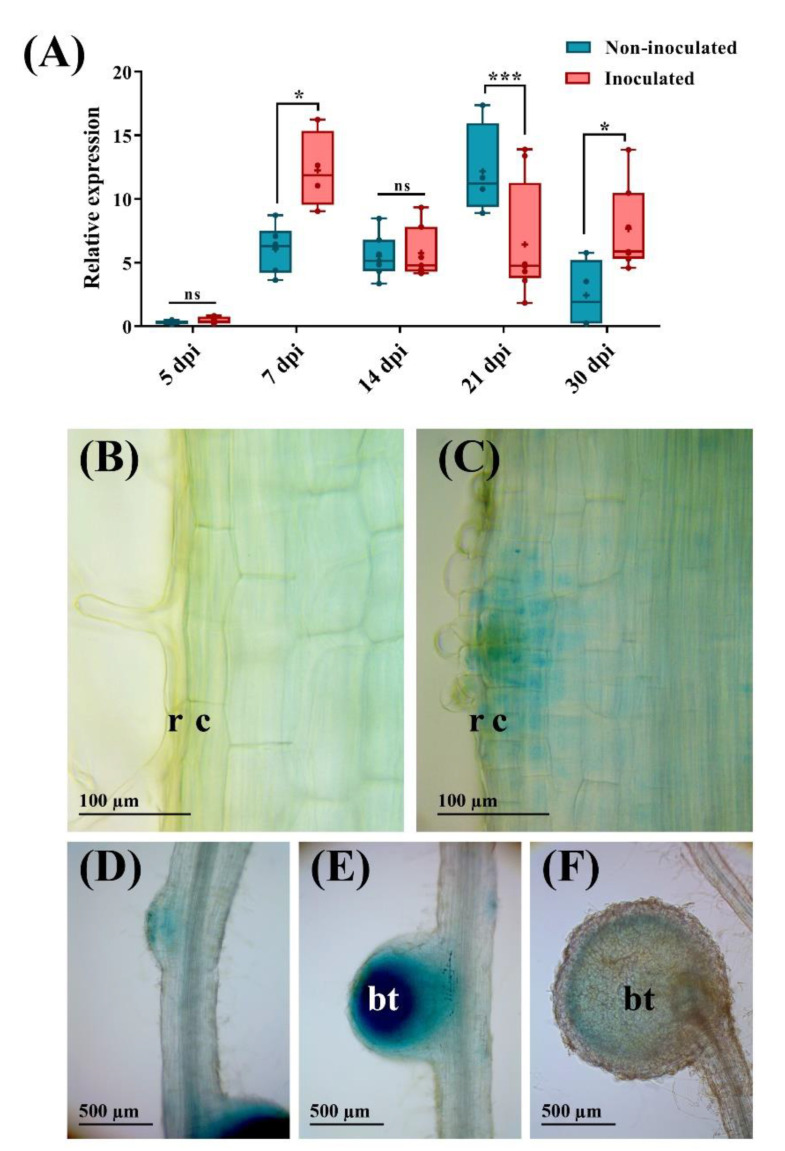
Expression profile analysis of *PvMT1A*. (**A**) Relative *PvMT1A* expression in *P. vulgaris* roots at 5, 7, 14, 21, and 30 dpi with *R. tropici* CIAT899 evaluated by qPCR. The elongation factor *EF1α* and *IDE* genes were used as endogenous reference genes to normalize expression levels. The blue bars represent non-inoculated roots, and the red bars represent roots inoculated with *R. tropici* at the indicated times. The top and bottom edges of the boxes delineate the first to third quartiles, the horizontal line within the box represents the median, and the whiskers indicate the smallest and largest outlier in the data set (*n* = 9). A non-parametric Mann–Whitney test was performed to evaluate significant differences, * *p* ≤ 0.05, *** *p* ≤ 0.001, and ns = no significant difference. (**B–F**) Promoter activity of *PvMT1A* visualized by GUS staining in non-inoculated (**B**) or inoculated (**C–F**) roots carrying p*PvMT1A*::GFP:GUS. (**B**) non-infected root hair, (**C**) curled root hair after rhizobial infection at 7 dpi, (**D**) nodule primordium at 30 dpi, (**E**) young nodule at 30 dpi, (**F**) mature nodule at 30 dpi. r, rhizodermis; c, cortex; bt, bacteroid tissue.

To verify the in silico data, we performed qPCR to analyze *PvMT1A* transcript levels in different *P. vulgaris* tissues: leaves, stems, root hairs, root tips, stripped roots (roots with the root hairs removed), roots inoculated with *R. tropici* (14 dpi) without nodules, nodules at 14 dpi, and non-inoculated roots at the same time point. As expected, the highest levels of *PvMT1A* expression were observed in both inoculated and non-inoculated roots and in root hairs and nodules (Figure S6). To further investigate the expression profile of *PvMT1A* during nodule symbiosis, we analyzed the transcript abundance of this gene during the early and late stages of nodulation in *P. vulgaris*. The expression pattern of *PvMT1A* was variable throughout the nodulation process (Figure 2A). During the early stages, *PvMT1A* was upregulated at 7 dpi, while during the late stages, it was downregulated at 21 dpi but upregulated again by 30 dpi. Taken together, these results strongly suggest that *PvMT1A* is involved in root nodule symbiosis in common beans.

To examine the space and time gene expression of *PvMT1A*, promoter activity of *PvMT1A* during rhizobial symbiosis was analyzed. We transformed *P. vulgaris* seedlings with the p*PvMT1A*::GFP:GUS construct and inoculated them with *R. tropici*. Based on the expression profile of *PvMT1A* observed during the nodulation process, we evaluated the promotor activity in transgenic roots at 7 and 30 dpi by GUS staining. We did not observe GUS activity in uninoculated or inoculated roots carrying the empty vector neither at 7 days nor 30 days (Figure S7A–F). At seven days, uninoculated roots carrying the p*PvMT1A*::GFP:GUS construct showed a basal GUS activity in root stele, primordia of lateral roots, and in the apical region of the main root (Figure S7G–I). In inoculated p*PvMT1A*::GFP:GUS roots (7 dpi), GUS activity was observed in divided cortical cells near to a deformed root hair, i.e., in the early nodule primordium (Figure 2C). In addition, the promoter maintained a basal activity in the primordia of lateral roots and increased its activity in the root stele and in the apical region of the main root (Figure S7J–L). At 30 dpi, GUS activity was observed in nodule primordia, the bacteroid tissue of young nodules, and in the periphery of the bacteroid tissue of mature nodules (Figure 2D–F). Furthermore, at this sampling time, GUS activity was also detected in the root stele and in the apical region of the lateral roots (Figure S7M,O). However, a low basal GUS activity was observed in the root stele of the lateral roots of the first order (Figure S7N). These observations suggest that *PvMT1A* expression increases in root stele and in the apical region of the main roots after inoculation with rhizobia during the early stages of infection, as well as during nodule morphogenesis.

### 2.3. PvMT1A Localizes to the Cytoplasm

**Figure 3:**
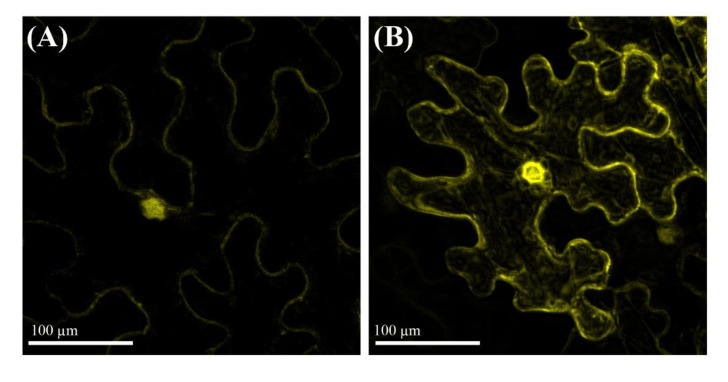
Subcellular localization of *Pv*MT1A. The localization of the *Pv*MT1A protein was monitored in *N. benthamiana* leaves infiltrated with the 35S::YFP construct as a control (**A**) or with 35S::YFP:*Pv*MT1A (**B**) by means of confocal microscope.

To examine the subcellular localization of PvMT1A, we transiently expressed *PvMT1A* in *N.* *benthamiana* leaves using *A. tumefaciens* strain CV3010 carrying the 35S::YFP:PvMT1A construct. In vivo images obtained by confocal microscope revealed fluorescence in the cytoplasm and cell periphery (Figure 3B), pointing to the cytoplasmic localization of PvMT1A. This result is in agreement with the structure, size, and composition of the predicted amino acid sequences of MTs [28] and also with reports where MT proteins have been localized to the cytosol in arabidopsis [29]. Furthermore, high fluorescence was also observed in the nucleus, suggesting nuclear localization as previously reported in mammalian cells in different studies during cell proliferation and development [30,31,32,33]. However, this finding should be taken with caution considering that PvMT1A is not under its own promoter, and we detected fluorescent background in the control nucleus, which could be generated by non-specific diffusion of YFP through nuclear pores [34].

### 2.4. Downregulation of PvMT1A Reduces Infection Events by R. tropici

To investigate the role of *PvMT1A* in the symbiosis of common bean with *R. tropici*, we performed RNA interference (RNAi) to target a specific region of its 3′UTR as described in the Materials and Methods. Transgenic roots were generated using two independent clones of *A. rhizogenes* K599 carrying *PvMT1A*:RNAi (C4 and C5). We assessed the effectiveness of the *PvMT1A*:RNAi constructs by qPCR analysis of *PvMT1A* expression in several independent transgenic plants under nodulation conditions. *PvMT1A* transcript levels in roots non-inoculated with *R. tropici* were 62% and 83% lower in *PvMT1A*:RNAi C4 compared to control transgenic roots at 7 and 14 dpi, respectively. *PvMT1A*:RNAi C5 reduced the transcript levels by 84% and 86% at 7 and 14 dpi, respectively (Figure S8A). A similar expression pattern was detected in inoculated roots at the same sampling times with a reduction in the transcript level around 78% and 70% at 7 and 14 dpi, respectively, in both RNAi clones (Figure S8B). To evaluate the effect of downregulation on the development of *P. vulgaris* roots, we examined transgenic roots of *PvMT1A*:RNAi plants non-inoculated at 7 days in pots. As shown in Figure S9A, the downregulation of *PvMT1A* did not affect the length of the main root, but the number of lateral roots was significantly reduced (Figure S9B).

**Figure 4:**
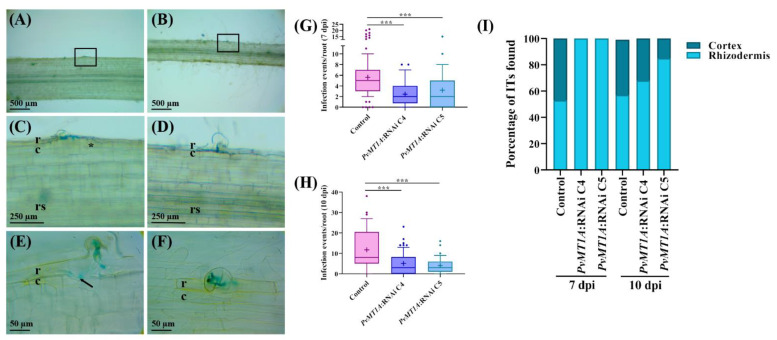
Analysis of infection events in control and *PvMT1A*-silenced transgenic roots detected by GUS staining. Representative images of infection events in control (**A**) and *PvMT1A*:RNAi C4 (**B**) transgenic roots inoculated at 7 dpi with *R. tropici*-*GUS*. Close-up of the marked area of panels (**A**,**B**) of root hair zones showing infection events in control (**C**) and *PvMT1A*:RNAi C4 (**D**) transgenic roots, respectively; IT of control (**E**) and *PvMT1A*:RNAi C4 (**F**). Cell divisions and IT invasion into cortical layers are shown with an asterisk and an arrow, respectively. The average number of total infection events was scored in control, *PvMT1A*:RNAi C4, and *PvMT1A*:RNAi C5 roots at 7 dpi (**G**) and 10 dpi (**H**) with *R. tropici*-*GUS*. The top and bottom edges of the boxes delineate the first to third quartiles, the horizontal line within the box represents the median, and the whiskers represent 10th and 90th percentiles in the data set (*n* = 15). A non-parametric Kruskal–Wallis test followed by Dunn’s multiple comparisons test was performed to evaluate significant differences *** *p* ≤ 0.001. Percentage of IT in control and *PvMT1A*:RNAi observed in root hairs and cortical cells (**I**). In (**C**–**F**), c, cortex; r, rhizodermis, and rs: root stele.

An analysis of infection events in transgenic roots inoculated with *R. tropici* harboring a *GUS* reporter gene (*R. tropici-GUS*) showed that the downregulation of *PvMT1A* reduced the total number of ITs by 50% and 75% at 7 and 10 dpi, respectively, compared to the control (Figure 4A–C,G,H). Importantly, at 7 dpi, 100% of the ITs observed in silenced roots were still within the root hairs, while 50% of those in control roots had reached the outer cortex zone. A similar reduction in IT progression at 10 dpi was observed in *PvMT1A*:RNAi transgenic roots, with less than 30% of the ITs detected in the outer cortex (Figure 4E). However, aborted ITs were not observed in silenced root hairs. Nevertheless, a reduction in IT progression was evident in *PvMT1A*:RNAi transgenic roots compared to the control (Figure 4F,I). These results indicate that *PvMT1A* contributes to *R. tropici* infection of hair cells and the outer cortex of common bean roots, leading to nodule formation.

To further explore the role of *PvMT1A* in rhizobial symbiosis in *P. vulgaris*, we examined the transcript levels of two early nodulin genes, *ENOD2* and *ENOD40*, in hairy roots carrying either the silencing construct *PvMT1A*:RNAi or the control. *ENOD2* and *ENOD40* transcript levels typically increase during cortical cell division in the early stages of nodule development [35]. Significantly higher *ENOD2* and *ENOD40* transcript levels were detected in control transgenic roots inoculated with *R. tropici* at 7 dpi compared to non-inoculated roots. However, no significant differences were observed between inoculated and non-inoculated *PvMT1A*:RNAi transgenic roots (Figure S10). Taken together, these results suggest that *PvMT1A* participates in rhizobial infection and IT progression in common beans.

### 2.5. Downregulation of PvMT1A Decreases Nodule Formation and Nitrogen Fixation

**Figure 5:**
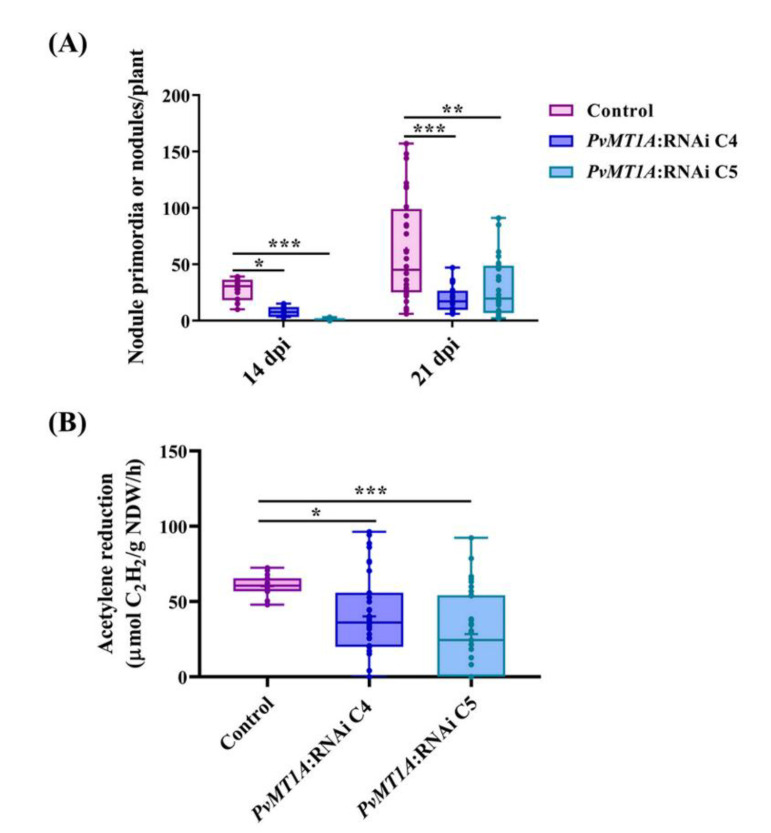
Measurement of nodulation capacity in control and *PvMT1A*-silenced transgenic roots. Total number of nodules in control, *PvMT1A*:RNAi C4, and *PvMT1A*:RNAi C5 hairy roots at 14 and 21 dpi (**A**). Nitrogenase activity in control, *PvMT1A*:RNAi C4, and *PvMT1A*:RNAi C5 transgenic roots inoculated with *R. tropici* at 21 dpi, as determined by an acetylene reduction assay (**B**). The top and bottom edges of the boxes delineate the first to third quartiles, the horizontal line within the box represents the median, and the whiskers represent the smallest and largest outlier in the data set (*n* = 30). A non-parametric Kruskal–Wallis test followed by Dunn’s multiple comparisons test was performed to evaluate significant differences, * *p* ≤ 0.05, ** *p* ≤ 0.01, *** *p* ≤ 0.001.

Since downregulating *PvMT1A* decreased IT formation and progression, we predicted that the total number of nodules would also decrease in these plants. To test this notion, we counted the number of nodules in *PvMT1A*:RNAi transgenic roots at 14 and 21 dpi with *R. tropici*. Compared to control roots, *PvMT1A*:RNAi roots showed an approximately 20% and 80% reduction in nodule formation at 14 and 21 dpi, respectively (Figure 5A). Furthermore, we estimated the nitrogen fixation rate in control and *PvMT1A*:RNAi transgenic roots at 21 dpi by measuring the rate of acetylene reduction. As shown in Figure 5B, *PvMT1A* downregulation caused a decrease in acetylene reduction (50%) compared to the control. These results reveal that nodule formation and nitrogenase activity are deficient in roots with downregulated *PvMT1A*.

To confirm the positive effect of *PvMT1A* on nitrogen fixation in *P. vulgaris*, we measured the transcript levels of the leghemoglobin gene in inoculated and non-inoculated transgenic roots. Leghemoglobin is a heme-containing protein responsible for maintaining the free oxygen concentration in the root nodule at a level that allows nitrogenase activity and respiration of both the host and the rhizobium [36]. In control transgenic roots, the transcript level of this gene increased after inoculation with rhizobia, as was expected (Figure S11). However, no significant changes were observed in *PvMT1A*:RNAi transgenic roots inoculated with *R. tropici* (14 dpi) compared to non-inoculated roots. These results confirm the notion that downregulating *PvMT1A* negatively affects nitrogen fixation during *P. vulgaris*-*R. tropici* symbiosis, affecting the leghemoglobin transcript levels, but more experiments are needed to elucidate the molecular mechanism that occurs between both genes.

### 2.6. Reactive Oxygen Species Production Is Modified in PvMT1A:RNAi Transgenic Roots

**Figure 6:**
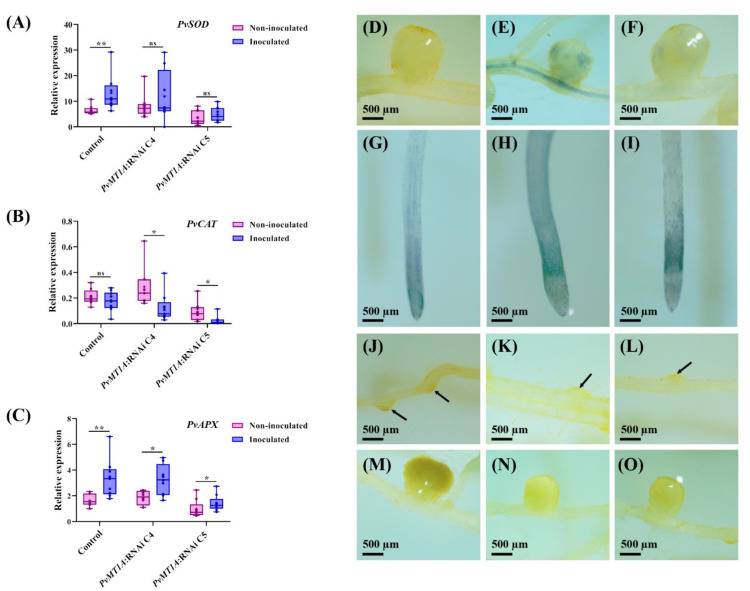
Expression levels of ROS gene markers and ROS production analysis in control and *PvMT1A*:RNAi transgenic roots. Relative expression profiles of *PvSOD* (**A**), *PvCAT* (**B**), and *PvAPX* (**C**) in control and *PvMT1A*:RNAi transgenic roots at 7 dpi with *R. tropici* evaluated by qPCR. The elongation factor *EF1α* and *IDE* genes were used as endogenous reference genes to normalize expression levels. The top and bottom edges of the boxes delineate the first to the third quartiles, the horizontal line within the box represents the median, and the whiskers represent the smallest and largest outlier in the data set (*n* = 9). (**A**) Non-parametric Mann–Whitney test was performed to evaluate significant differences, * *p* ≤ 0.05, ** *p* ≤ 0.01, and ns = no significant difference. In panels (**D**−**O**), the visualization of O_2_^−^ and H_2_O_2_ in representative roots at 14 dpi with *R. tropici* CIAT899 by NBT and DAB staining are shown, respectively. The blue color indicates the presence of O_2_^−^ in the tissue, and the brown color indicates the presence of H_2_O_2_ in the tissues. Typical NBT-stained nodules of control transgenic roots (**D**), *PvMT1A*:RNAi C4 (**E**), and *PvMT1A*:RNAi C5 (**F**). Representative samples of control transgenic roots (**G**), *PvMT1A*:RNAiC4 (**H**), and *PvMT1A*:RNAiC5 (**I**) stained with NBT. DAB staining of representative early nodule primordia of the control (**J**), *PvMT1A*:RNAi C4 (**K**), and *PvMT1A*:RNAi C5 (**L**). Arrows in (**J**–**L**) indicate the presence of H_2_O_2_ in the nodule primordia. Typical nodules of control (**M**), *PvMT1A*:RNAi C4 (**N**), and *PvMT1A*:RNAi C5 (**O**) transgenic roots stained with DAB.

MTs act as reactive oxygen species (ROS) scavengers in plants [26]. To better understand the molecular mechanisms underlying the regulatory role of *PvMT1A* in *P. vulgaris-R. tropici* symbiosis, we quantified the expression levels of genes related to ROS production by qPCR. Specifically, we quantified the transcript levels of *PvRbohA, PvRbohB, PvSOD*, *PvCAT,* and *PvAPX* genes in both *PvMT1A*:RNAi and control transgenic roots at 7 dpi. *PvRbohA* and *PvRbohB* are involved during common bean-rhizobium symbiosis [37,38], while *SOD*, *CAT*, and *APX* are important regulators of ROS homeostasis during fabacean-rhizobia symbiosis [39]. Interestingly, we observed significant changes in the expression profile of *PvRbohA* in control transgenic roots inoculated with *R. tropici* compared to non-inoculated roots (Figure S12A). Moreover, no significant differences were observed in the expression profile of *PvRbohB* in control transgenic roots or in *PvMT1A*:RNAi inoculated or non-inoculated roots (Figure S12B). However, in control roots, inoculation with rhizobia induced an increase in *PvSOD* transcript accumulation, while in silenced roots, no significant changes were observed (Figure 6A). Furthermore, there were no significant changes in *PvCAT* transcript levels in inoculated control roots compared to non-inoculated roots. Meanwhile, *PvCAT* was downregulated in inoculated *PvMT1A*:RNAi transgenic roots compared to inoculated control roots (Figure 6B). In addition, an increase in *PvAPX* transcript accumulation was observed both in the control inoculated with rhizobia and in the roots of *PvMT1A*:RNAi (Figure 6C).

Due to the downregulation of *PvMT1A*, the expression of *PvCAT* was reduced and appeared to impair the rhizobia-induced upregulation of *PvSOD*. We analyzed the levels of O_2_^−^ and H_2_O_2_ in control and *PvMT1A*:RNAi transgenic roots. NBT staining to detect O_2_^−^ in transgenic roots at 14 dpi showed accumulation of O_2_^−^ in vascular tissues and nodules of *PvMT1A*:RNAi roots (Figure 6E,F) and in the region very close to the apex of the roots (Figure 6H,I), while in control roots, O_2_^−^ accumulates only in the apical region of the root and not in nodules (Figure 6D,G). On the other hand, DAB staining revealed a reduction in H_2_O_2_ levels in *PvMT1A*:RNAi root nodule primordia and nodules compared to the control (Figure 6J–O). H_2_O_2_ accumulation was also observed in nodule primordia and mature nodules in control roots (Figure 6J,M). Taken together, these results suggest that the deregulation of *PvMT1A* affected the O_2_^−^ metabolism during rhizobial symbiosis in *P. vulgaris*.

## 3. Discussion

Plant MTs genes comprise a heterogeneous family of ubiquitous metal ion-binding proteins that are grouped into four classes [26]. These proteins are characterized by large numbers of cysteine residues, which constitute their typical protein motifs [12]. MTs participate in the regulation of cell growth and proliferation, protection against toxic metals and metal homeostasis, ROS scavenging, and plant responses to attack by pathogens [26]. However, despite their wide variety of functions, the role of MTs in symbiotic associations such as nodule symbiosis has been poorly studied. Here, we demonstrated that *PvMT1A* participates in the early and late stages of root nodule symbiosis in common beans.

Our in silico analysis identified six *MT* genes in common beans (*PvMT*), all of which showed a conserved structure with a single intron. This characteristic has also been observed in most of the *MT* genes of several nonfabacean dicotyledonous species, including *A. thaliana* and several species of *Brassica* [40]. By contrast, in rice, a monocotyledonous species, most of the *MT* genes contain two introns [41]. These findings indicate that the structure of *MT* genes is highly conserved within each species but not between the dicots and monocots analyzed here. Plant MTs are thought to have arisen before the separation of monocots and dicots during evolution [42]. However, our phylogenetic analysis indicated that most monocot MTs, but only a minority of fabacean and *A. thaliana* MTs, are clustered in the MT2 clade. Together, these findings suggest that, although all four MT clades are distributed throughout the angiosperms and evolved before the divergence of monocots and dicots, gene structure and close phylogenetic relationships are conserved within each group. We identified 13 *MT* genes in the rice genome, more than the 11 *MT* genes found in previous studies [41,43]. Likewise, our findings for arabidopsis differ from those of [40]: we do not consider the *AtMT1B* gene to be a member of the MT family because the corresponding protein lacks cysteine motifs.

*MT* genes are expressed in a tissue-specific manner in plants. Members of the MT1 class are mainly expressed in roots [12,13]. In agreement with this finding, the highest transcript level for *PvMT1A* was observed in uninoculated roots at 14 days, followed by inoculated roots at 14 dpi (Figure S6). However, *PvMT1A* was also highly expressed in root hairs in 2-day-old uninoculated roots and nodules at 14 dpi (Figure S6); moreover, after rhizobial inoculation, *PvMT1A* transcript levels increased at 7 and 30 dpi (Figure 2A). In particular, the expression profile of *PvMT1A* observed during the early stages of rhizobial symbiosis could indicate that PvMT1A participates in the infection process. Supporting this idea are the findings that the *PvMT1A* promoter is active in the cortex and rhizodermis at infection sites and that downregulating *PvMT1A* reduced both the number of ITs and the proportion of ITs that had entered the cortex compared to control roots at 7 and 10 dpi (Figure 4). This reduction in the number of ITs could be related to the deregulation of *ENOD2* and *ENOD40* (Figure S10); these genes are known to be involved in the early stages of nodulation. In previous studies, both genes were found to be upregulated in response to rhizobial inoculation during cortical cell divisions in the early stages of nodule organogenesis [35,44]. On the other hand, the induction of *PvMT1A* transcript accumulation observed in roots at 30 dpi suggests that this gene is likely involved in the characteristic oxidative stress that occurs during nodule senescence [45,46]. In addition, the high expression of *PvMT1A* in non-inoculated roots at different time points also suggests a role of this gene in the root development, which is supported by the reduction in lateral root development (Figure S9). Nonetheless, further experiments are needed to confirm this notion.

The average number of nodules was lower in *PvMT1A*-silenced transgenic roots than in control roots at 14 and 21 dpi (Figure 5A); this was expected, considering the reduction in the number of ITs in *PvMT1A*:RNAi roots. Remarkably, in the few nodules in *PvMT1A*:RNAi transgenic roots, a 50% reduction in nitrogen fixation capacity was observed (Figure 5B). Rhizobia are obligate aerobic microorganisms that require oxygen for their metabolism, but they also need the level of O_2_ to be precisely regulated to protect the nitrogen fixation process. Leghemoglobin is one of the key players required for efficient nitrogenase activity, limiting free O_2_ levels while also maintaining an adequate O_2_ flow to support vigorous respiration by bacteroids and host mitochondria [47]. In the current study, the transcript accumulation of leghemoglobin in silenced roots did not increase as it did in control roots at 14 dpi (Figure S12), pointing to a transcriptional relationship between *PvMT1A* and leghemoglobin during root nodule symbiosis in *P. vulgaris*. The *nif* genes encoding the nitrogenase complex in rhizobia are known to be negatively regulated by oxygen [48,49]. Therefore, the low nitrogenase activity in silenced root nodules could be due to the lack of regulation of oxygen levels in the nodules.

ROS are continuously generated as normal by-products of aerobic metabolism [50]. ROS-mediated signaling plays an essential role in both the early and late stages of nodule symbiosis [51,52,53]. Accordingly, at 7 dpi, rhizobial inoculation increased *PvRbohA*, *PvSOD*, and *PvAPX* transcript accumulation in transgenic control roots, but this increase did not occur for *PvRbohA* and *PvSOD* in inoculated *PvMT1A*:RNAi roots (Figure 6, Figure S11). Moreover, *PvCAT* expression decreased in inoculated *PvMT1A*:RNAi roots at 7 dpi (Figure 6B). These findings suggest that the loss-of-function of *PvMT1A* prevents rhizobia-dependent induction of *PvRbohA* and *PvSOD* expression and leads to reduced *PvCAT* expression during nodule symbiosis but does not affect *PvAPX* expression. Notably, the expression of *PvRbohA* and *PvRbohB*, genes associated with the production of O_2_^−^ in common bean roots [37,38], responded differentially to rhizobial inoculation in *PvMT1A*:RNAi or control roots. Several *Rbohs* have also been found to be involved in rhizobia symbiosis. For instance, downregulation of *PvRbohA* reduced rhizobial infection and nitrogenase activity [38], as well as the *PvSOD* and *PvCAT* expression in inoculated and non-inoculated roots with *R. tropici* [38]. Furthermore, the loss-of-function of *MtRbohA* also reduced nitrogen fixation activity [53]. Therefore, our results suggest that *PvRbohs* are related to *PvMT1A* in ROS metabolism during rhizobial symbiosis through *PvRbohA*.

The high respiratory activity required to support nitrogen fixation, along with oxidation of leghemoglobin and various other molecules within the nodules, leads to high rates of ROS production [39]. Taking this into account, we analyzed the qualitative accumulation of O_2_^−^ and H_2_O_2_ in inoculated *PvMT1A*:RNAi and control transgenic roots. The downregulation of *PvMT1A* affected O_2_^−^ distribution (Figure 6D–I) and reduced H_2_O_2_ levels in the roots (Figure 6J–O). This result could indicate that the downregulation of *PvMT1A* not only prevents the induction of rhizobia-dependent *PvSOD* expression but also the enzymatic activity of *Pv*SOD. In this sense, it is important to point out that H_2_O_2_ can be produced from O_2_^−^ through the enzymatic activity of SOD and can also play a different role depending on its concentration. Therefore, the balance between the enzymatic activity of SOD and H_2_O_2_-scavenging enzymes is essential to maintain O_2_^−^ and H_2_O_2_ at adequate levels [54]. The fact that the expression of *PvSOD* was not induced by rhizobium inoculation in the roots of *PvMT1A*:RNAi plants together with the low presence of H_2_O_2_ levels in these silenced roots, as occurred in the control roots, could be a mechanism to prevent cellular damage caused by the high H_2_O_2_ levels.

One of the main mechanisms for the decrease in H_2_O_2_ is the activity of CAT [54]; intriguingly, we observed a reduction in the expression of *PvCAT* in *PvMT1A*:RNAi plants. In addition, our results showed that *APX* expression was not affected by *PvMT1A* silencing, suggesting that this H_2_O_2_-scavenging enzyme is likely involved in H_2_O_2_ homeostasis during nodule symbiosis independently of *PvMT1A*. However, APX is known to participate in the ascorbate-glutathione (GSH) pathway, an important antioxidant mechanism in nodules, and is highly expressed in nodules [39]. On the other hand, we hypothesize that the reduction in the number of lateral roots produced by the downregulation of *PvMT1A* (Figure S9B) could be due, at least partially, to the reduction in H_2_O_2_. The participation of H_2_O_2_ in the development of lateral roots has been previously demonstrated. For instance, treatment with H_2_O_2_ increased the density of lateral roots and induced their emergence of the double mutants arabidopsis *aux1* and *lax3*, which lack lateral roots [55]. Moreover, it has been shown that H_2_O_2_ is required for the formation of lateral roots in tomato (*Solanum lycopersicum* L.) [56] and alfalfa (*Medicago sativa* L.) seedlings [57]. Changes in the accumulation of *PvMT1A* transcripts in non-inoculated roots (Figure 2A) could be related to the functions of *PvMT1A* in root development through the regulation of ROS balance. Root architecture has been shown to be controlled by the root stem cell niche in the apical region, requiring a fine-tuning regulation of O_2_^−^ and H_2_O_2_ levels for their division and differentiation, respectively [58,59].

To date, MTs have been shown to be key regulators of ROS homeostasis in response to several abiotic stresses [17,60] and in plant-pathogen interactions [16]. Our findings provide compelling experimental evidence highlighting the role of MTs in rhizobial infection and in nodule development during the mutualistic interaction between common bean and *R. tropici*. Our results suggest that MTs participate in the regulation of ROS levels. However, more experimental approaches are needed to better understand the specific mechanism by which *PvMT1A* participates in the regulation of rhizobia-symbiosis establishment.

## 4. Materials and Methods

### 4.1. Database Search and Gene/Protein Sequence Analysis

To identify the genes encoding MTs in the common bean genome, a search was performed in the Phytozome 12.1.6 database (https://phytozome.jgi.doe.gov; [22] Accessed on 4 February 2019). The Phvul.L001645.1 sequence previously identified by transcriptomic analysis by the authors of [10] and the sequences of the seven arabidopsis *MT* genes [12,25] were used as queries. The expression profiles of the *MT* genes from common bean were retrieved from the Gene Expression Atlas (PvGEA, http://plantgrn.noble.org/PvGEA/; [27] Accessed on 11 February 2019). Domain identification and functional annotation were performed using Blast2GO software [61] with the InterPro and UniProt databases. Conserved motifs in the full-length amino acid sequences were identified using the Multiple Expectation maximizations for Motif Elicitation (MEME) tool version 5.2.0 [62]. The theoretical molecular weight and isoelectric point of the PvMTs were calculated using the ExPASy web server (https://web.expasy.org/compute_pi/; [63] Accessed on 6 July 2019).

### 4.2. Chromosomal Localization and Gene Structure Analysis

The chromosomal positions of common bean *MT* genes were identified using PhenoGram Plot (https://visualization.ritchielab.org/phenograms/plot; [64] Accessed on 20 July 2019). Gene duplication and exon-intron gene structure data were obtained from the Phytozome 12.1.6 database, and analysis of gene structure was performed using Gene Structure Display Server 2.0 (http://gsds.gao-lab.org/Gsds_about.php; [65] Accessed on 21 July 2019).

### 4.3. Phylogenetic Analysis of MTs

To examine the evolutionary relationships of *P. vulgaris* MTs, a phylogenetic analysis was performed using the amino acid sequences of MTs from several fabaceans (*Glycine max* (L.) Merr, *Lotus japonicus* L., and *Medicago truncatula* Gaertn.), nonfabacean dicotyledonous species (*Arabidopsis thaliana* (L.) Heynh, *Populus trichocarpa* Torr. and A. Gray ex. Hook., *Vitis vinifera* L., and *Amaranthus hypochondriacus* L.), and monocotyledonous species (*Oryza sativa* L., *H. vulgare* L., *Zea mays* L., and *Sorghum bicolor* (L.) Moench). Protein sequences were aligned with the MUSCLE algorithm and manually edited using MEGA version X [66] to eliminate misaligned sequences. The phylogenetic tree was reconstructed with the IQ-TREE algorithm version 1.6.12 [67] using the maximum-likelihood method based on the Dayhoff model with 1000 bootstraps.

### 4.4. Growth Conditions of Wild-Type Plants

Seeds of *P. vulgaris* cv. Negro Jamapa (purchased from the company “El Caudillo” Sociedad de Producción Rural Morelos, Mexico) were surface sterilized as described by the authors of [68] and incubated in a germination chamber for 48 h at 28 °C under non-light conditions and 30% humidity. At two days post germination (2 dpg), the seedlings were planted in pots with sterile vermiculite and inoculated or not (control plants) with *R. tropici* CIAT 899 [69] at an OD_600_ of 0.05. Inoculated plants were watered with nitrate-free B and D solution [70], and control plants (non-inoculated) were treated with KNO_3_ (10 mM) to avoid rhizobial infection. In order to analyze the tissue-specific expression of *PvMT1A* different tissues: leaves, stems, root hairs, 2 dpg radicle tips and stripped radicle (radicle with the root hairs removed), roots inoculated with *R. tropici* (14 dpi) without nodules, nodules at 14 dpi, and non-inoculated roots at the same time point were collected. In addition, the roots of 5, 7, 14, 21, and 30 dpi with *R. tropici* CIAT899 and the control roots used for the quantification of *PvMT1A* during early and late stages of nodulation were grown in the same conditions described earlier. All the tissues were collected and frozen in liquid nitrogen and then stored at −75 °C until RNA extraction. Subsequently, the reverse transcription reaction and the qPCR analysis were performed. Three biological replicas were made for each sampling time, and three independent plants were used in each biological replication (*n* = 9).

### 4.5. Plasmid Construction

To construct the vector for the subcellular localization of PvMT1A, the coding sequence (CDS) of *PvMT1A* was amplified with specific primers (Table S4) and cloned into pENTR^TM^/D-TOPO^®^ (Invitrogen, Life Technologies, Carlsbad, CA, USA). The resulting vector, pENTR/D-TOPO-*PvMT1A*, was recombined with the destination vector pEARLEY104 (CD3-686 stock provided by Craig Pikaard and Keith Earley), generating the 35S::YFP:*Pv*MT1A construct. The empty vector pEARLEY104 was used as a control. Cloning and recombination reactions were performed by Gateway Technology (Invitrogen Gateway cloning technology). To analyze the promoter activity of *PvMT1A*, an 1884-bp fragment upstream of the translation start site was amplified from common bean genomic DNA using specific primers (Table S4) and cloned into the pJET1.2/blunt vector (Thermo Fisher Scientific Baltics UAB, Vilnius, Lithuania). This vector was digested with HindIII/XhoI (Invitrogen, Life Technologies, Carlsbad, CA, USA), and a fragment containing the promoter region (1884 bp) was extracted and inserted into the destination vector pCeSar, which was digested with HindIII/SalI (Invitrogen, Life Technologies, Carlsbad, CA, USA). The pCeSar vector was developed and kindly donated by Marco A. Juárez-Verdayes, Ph.D., a former member of our group. This vector contains the reporter genes *β*-glucuronidase (*GUS*) and green fluorescent protein (*GFP*) in transcriptional fusion with a nuclear localization signal (NLS) (Figure S13). The empty pCesar vector was used as a control.

The construct for *PvMT1A* RNAi silencing (*PvMT1A*:RNAi) was generated by cloning 144 bp from the 3’UTR of *PvMT1A*, which was amplified using common bean root cDNA as a template and specific primers (Table S4). The fragment was cloned in pENTR^TM^/D-TOPO^®^, and the resulting construct was recombined with the binary vector pTDT-DC-RNAi [71]. Cloning and recombination reactions were performed by Gateway Technology (Invitrogen Gateway cloning technology). The correct orientation of the inserted fragments in the resulting construct, *PvMT1A*:RNAi, was verified by PCR and sequencing. As a control, the pTdT-SAC vector was used, which carries a truncated and irrelevant sequence of *A. thaliana* pre-mir159 [37].

### 4.6. Subcellular Localization of PvMT1A

To determine the subcellular location of PvMT1A, transient expression was carried out in leaves of *Nicotiana benthamiana* Domin (collection of seeds from our laboratory). Four-week-old *N. benthamiana* leaves were infiltrated with 1 mL of infiltration buffer (2 µM Na_2_HPO_4_, 50 mM MES, 10 µM acetosyringone, and 15 mM sucrose) containing *Agrobacterium tumefaciens* strain CV3010 (provided by Prof. Alejandra Covarrubias, Ph.D., group, Morelos, Mexico) (OD_600_ 0.05; [72]) transformed with the 35S::YFP:PvMT1A construct. This construct contains the CDS of *PvMT1A* fused to the Yellow Fluorescent Protein (YFP) coding sequence under the control of the 35S promoter. After four days, the infiltrated leaves were cut, mounted on slides with water, and inspected using a confocal microscope (Olympus FV100, Olympus Corporation, Tokio, Japan).

### 4.7. Generation of Composite Plants

Common bean seedlings of 2 dpg were infected with *Agrobacterium rhizogenes* strain K599 [73], carrying the construct for promoter activity analysis (p*PvMT1A*::GFP:GUS) or the construct for RNAi silencing (*PvMT1A:*RNAi). As a control, composite plants carrying the respective control vector were generated. All composite plants were obtained as described earlier [68] with some modifications [74] under controlled conditions at 28 °C with 16/8 h light/dark and 30% humidity. To confirm the presence of the fluorescent reporter gene and to eliminate non-transformed roots, hairy roots were observed using an epifluorescence microscope (Olympus SZX2-ILLB, Olympus Corporation, Tokio, Japan). Composite plants were potted in sterile vermiculite and inoculated or not with *R*. *tropici* CIAT899 (OD_600_ 0.05) or *R. tropici*-*GUS* (OD_600_ 0.05) for further analysis.

To validate the silencing efficacy of the *PvMT1A*:RNAi constructs, by qPCR analysis, *PvMT1A*:RNAi and pTdT-SAC (control) roots were sampled at 7 and 14 dpi with *R. tropici* CIAT899. To quantify the accumulation of ascorbate peroxidase (*APX*, Phvul.011G071300.1), a Cu-Zn superoxide dismutase (*PvSOD*, Phvul.006G097000.1), a catalase (*PvCAT*, Phvul.001G001000.1), *PvRbohA,* and *PvRbohB*, *PvMT1A*:RNAi, and control roots were collected at 7 dpi with *R. tropici*. This protocol was also used to quantify the accumulation levels of *PvENOD2* (Phvul.002G259604.1) and *PvENOD40* (Phvul.002G064166.1) gene transcripts. These same transgenic roots sampled at 14 dpi were used for the quantification of leghemoglobin gene (Phvul.007G142600.1) transcripts. All root samples were obtained from three independent plants in two or three biological replicas (*n* = 6–9) and stored at −75 °C until RNA extraction. The quantification of the accumulation of transcripts of all these genes was carried out by qPCR analysis.

### 4.8. RNA Extraction and qPCR Assays

RNA extraction from frozen roots was performed using TRIzol Reagent (Ambion^®^, Life Technologies^TM^, Carlsbad, CA, USA) according to the manufacturer’s instructions. The integrity of the RNAs was verified by agarose gel electrophoresis (1%), and RNA concentration was measured on a NanoDrop 2000/200c (Thermo Scientific, Waltham, MA, USA). Total RNA was incubated with RNase-free DNase (10 U/µL, Roche, Mannheim, Germany) at 37 °C for 30 min to remove DNA contamination. Complementary DNA (cDNA) synthesis was performed from 200 ng/μL of DNA-free RNA using RevertAid Reverse Transcriptase (200 U/μL, Thermo Scientific, Waltham, MA, USA) according to the manufacturer’s instructions. qPCR assays were performed using Maxima SYBR Green/ROX qPCR Master Mix (2X) (Thermo Scientific, Waltham, MA, USA) on a qPCR system (QuantStudio 5; Applied Biosystems, Waltham, MA, USA) as follows: 95 °C for 10 min, 30 cycles of 95 °C for 15 s, and 60 °C for 60 s. The primer sequences used for qPCR are listed in Table S4. The 2^−ΔΔCT^ method was used to calculate the relative abundance of transcript for each gene using two reference genes, elongation factor 1α (*EF1α*, Phvul.004G075100.1) and insulin-degrading enzyme (*IDE*, Phvul.001G133200.1) for normalization, which were previously described and used by our group [37,75]. The samples for qPCR were obtained from three independent plants with two or three biological replicates and three technical repeats per sample.

### 4.9. Analysis of PvMT1A Promoter Activity

To analyze *PvMT1A* promoter activity, GUS activity was detected in inoculated or non-inoculated hairy roots expressing p*PvMT1A*::GFP:GUS or the empty vector pCeSar at 7 dpi. GUS activity was also detected in inoculated (30 dpi) hairy roots expressing both p*PvMT1A*::GFP:GUS or the empty vector. GUS activity was detected as described earlier [76].

### 4.10. Phenotypic Analysis of PvMT1A:RNAi plants

#### 4.10.1. Measurement of Infection Events and Quantification of Nodule Number

Hairy roots expressing the silencing construct (*PvMT1A*:RNAi) or the control vector (pTdT-SAC), inoculated with *R. tropici*-*GUS* (OD_600_ 0.05; [77]) were collected at 7, 10, 14, and 21 dpi of 101 roots from 7 plants for control plants, 38 roots from 5 plants for *PvMT1A*:RNAi C4, and 36 roots from 4 plants for *PvMT1A*:RNAi C5. Similarly, at 10 dpi, the number of ITs was quantified of 59 roots from 11 plants for the control, 74 roots from 13 plants for *PvMT1A*:RNAi C4, and 49 roots from 9 plants for *PvMT1A*:RNAi C5. The IT advancement through root hairs and root cortex cells was analyzed by GUS staining. The total number of ITs observed at 7 or 10 dpi was considered as 100% of ITs at each time point, and those ITs that were observed at the level of the cortex or rhizodermis level (the ITs began to grow in the root hair from the bottom of the trichoblast) were divided into both categories giving the 100% between them when added. While the number of nodules at 14 and 21 dpi of 30 plants in a total of three biological replicates was quantified.

#### 4.10.2. Nitrogen Fixation Assays

Nitrogenase activity was determined by measuring acetylene reduction [78]. Transgenic roots nodulated with *R. tropici* CIAT899 (21 dpi) of 30 plants per condition were placed in vials (160 mL). After sealing the vials with rubber stoppers, 2 mL of air was removed with a syringe, and the same amount of acetylene was injected into the vials. The samples were incubated for three hours at room temperature, and ethylene production was measured in a gas chromatograph (Varian model 3300) [79]. Subsequently, the nodules were removed from the root and dehydrated at 60 °C for five days to obtain the dry weight. Nitrogenase activity was expressed as µmol of ethylene h^−1^ nodule dry weight^−1^ (NDW).

#### 4.10.3. Analysis of Reactive Oxygen Species Production

Transgenic roots carrying the *PvMT1A*:RNAi and pTdT-SAC constructs were inoculated with *R. tropici* CIAT899; at 14 dpi, the roots were transferred to demineralized water and stained with nitroblue tetrazolium (NBT) or 3,3′-diaminobenzidine (DAB) to detect O_2_^−^ or H_2_O_2_, respectively. For O_2_^−^ detection, roots were incubated in 50 mM NaH_2_PO_4_ (pH = 7.5) with 1% NBT for 1 h in the dark. The root tissue was rinsed with 96% ethanol for 1 h, rehydrated (40 to 10% ethanol), and placed on slides containing 50% glycerol. For DAB staining, roots were incubated in demineralized water and acidified with a HCl solution (pH = 3.8) containing 1% DAB for 2 h in the dark. Subsequently, the roots were incubated in 96% boiling ethanol for 10 min, rehydrated, and placed on slides as described for NBT staining. In addition, the accumulation of transcripts of a superoxide dismutase, Cu-Zn *SOD*, a catalase, *CAT*, a cytosolic ascorbate peroxidase, *APX,* and a *PvRbohB* was analyzed in the transgenic control and in *PvMT1A*:RNAi roots at 7 dpi with *R. tropici*, see details in Section 4.7.

### 4.11. Statistical Analysis

Statistical analysis of the transcript accumulation of *PvMT1A* in wild-type roots and the accumulation of *PvRbohB*, *PvAPX, PvSOD*, *PvCAT*, *ENOD2*, *ENOD40*, and leghemoglobin genes in transgenic roots was performed using the non-parametric Mann–Whitney test. Data on transcript accumulation of *PvMT1A* in different *P. vulgaris* tissues, root development, infection events, number of nodules, and nitrogenase activity were analyzed using the Kruskal–Wallis test followed by Dunn’s multiple comparisons. All statistical tests were carried out with the statistics software GraphPadPrism version 8.0.2(263) (San Diego, CA, USA, www.graphpad.com, accessed on 23 December 2021).

## 5. Conclusions

This research describes the key role of an *MT* gene in early rhizobial infection and organogenesis of nodules in *P. vulgaris*. We have provided evidence demonstrating that the *PvMT1A* gene is required for the proper development and progression of ITs during the early stages of nodulation. Furthermore, *PvMT1A* plays a crucial role in nodulation and nitrogen fixation capacity. The data presented here suggest that *PvMT1A* participates in ROS homeostasis during rhizobial symbiosis in *P. vulgaris*.

## Supplementary Materials

The following are available online at https://www.mdpi.com/article/10.3390/ijms23031491/s1.
