# Supplementary material for: Metallothionein1A Regulates Rhizobial Infection and Nodulation in Phaseolus vulgaris
Source: Int J Mol Sci. 2022 Jan 27;23(3):1491. doi: 10.3390/ijms23031491 (PMC8836284; doi:10.3390/ijms23031491)
Supplement: Supplementary file 1 [file ijms-23-01491-s001.zip › Supplementary Mterial/CQ_Supplementary_Material.pdf]

Supplementary Material

Metallothionein1A Regulates Rhizobial Infection and Nodulation in *Phaseolus vulgaris*

Citlali Fonseca-García, Claudia Marina López-García, Ronal Pacheco, Elisabeth Armada, Noreide Nava, Rocío Pérez-Aguilar, Jorge Solis-Miranda, and Carmen Quinto

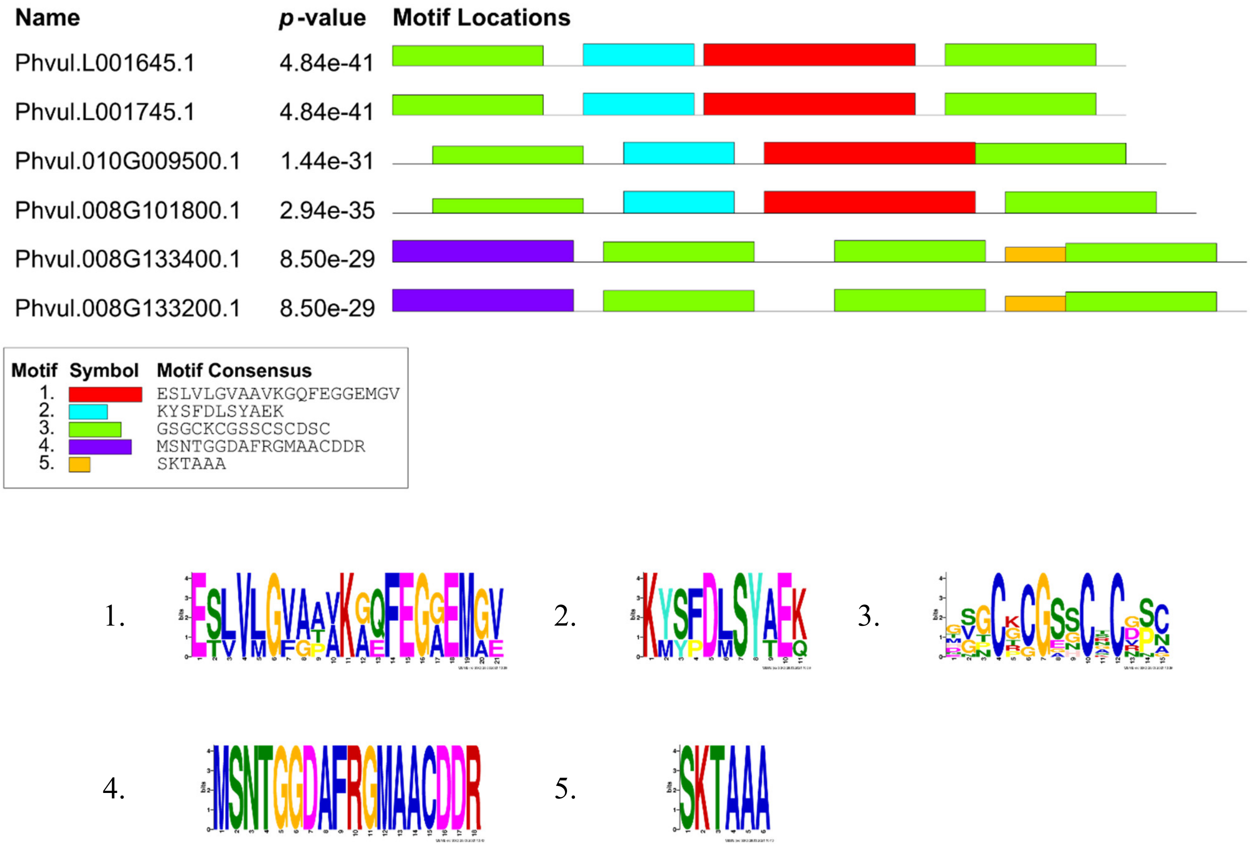

Figure S1. Functional motifs conserved in *Pv*MTs. Schematic representation of the motifs identified in *Pv*MT amino acid sequences. Significantly overrepresented motifs are shown by bars at their predicted positions. The logos of the identified overrepresented motifs are numbered as shown in the legend. MEME software was used to identify significantly overrepresented motifs.

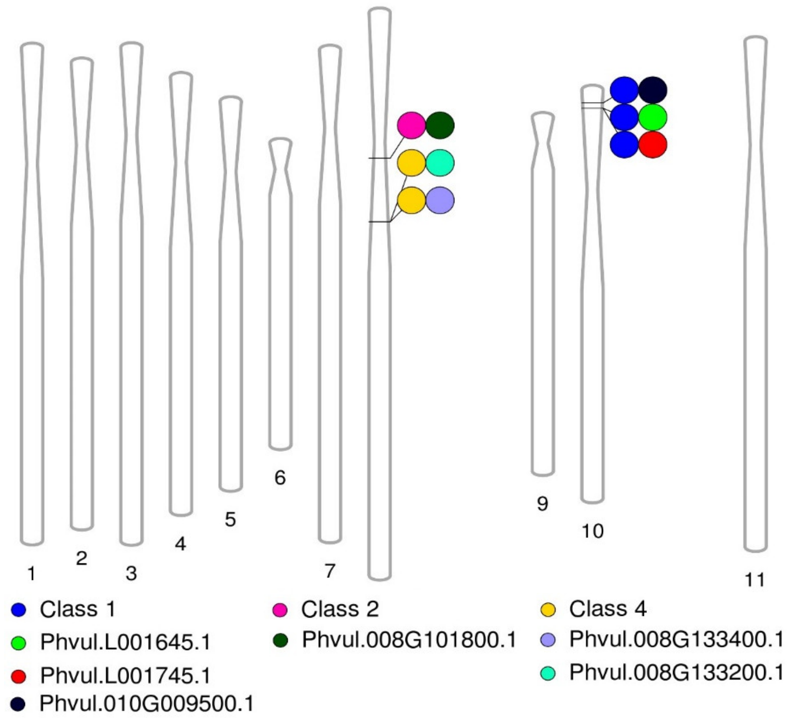

Figure S2. ***In silico*** **mapping of the** ***PvMT*** **gene loci.** Chromosomal locations of the *MT* genes in *P. vulgaris*. Colored dots indicate the chromosomal locations of the genes.

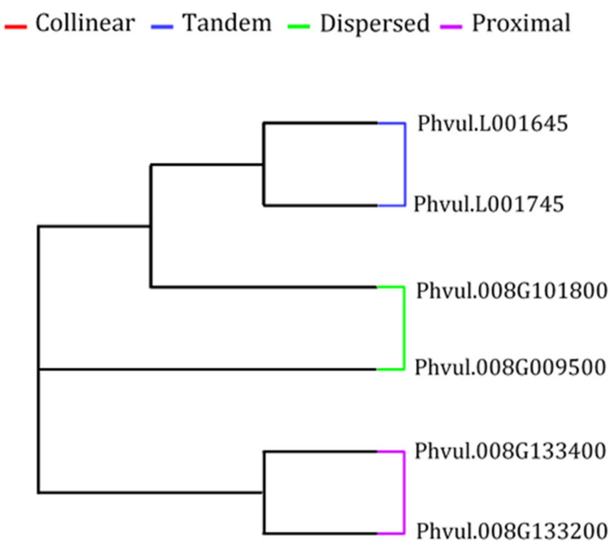

Figure S3. Duplication analysis of *PvMT* genes. Schematic representation of the duplication events in the six *MT* genes identified in *P. vulgaris*. MCScan software was used to analyze duplication events between *PvMT* sequences.

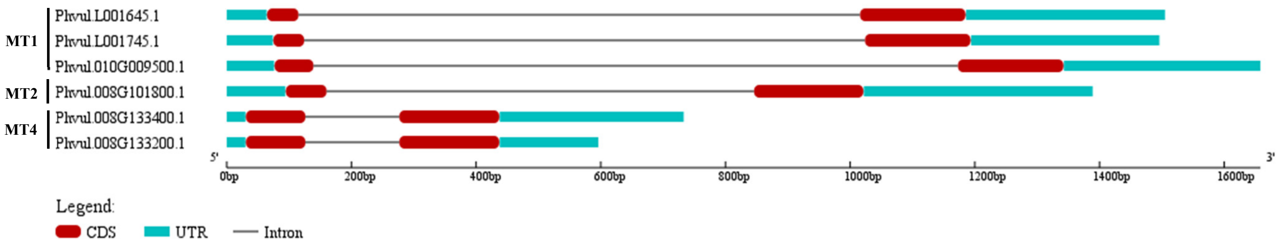

Figure S4. Gene structures of *PvMT*. Exon-intron compositions of the *MT* gene sequences of *P. vulgaris*. The MT classes on the left indicate the corresponding sequences for each class. Exons (CDS), introns, and untranslated regions (UTR) are represented according to the key. Gene Structure Display Server was used for the analysis.

(A)

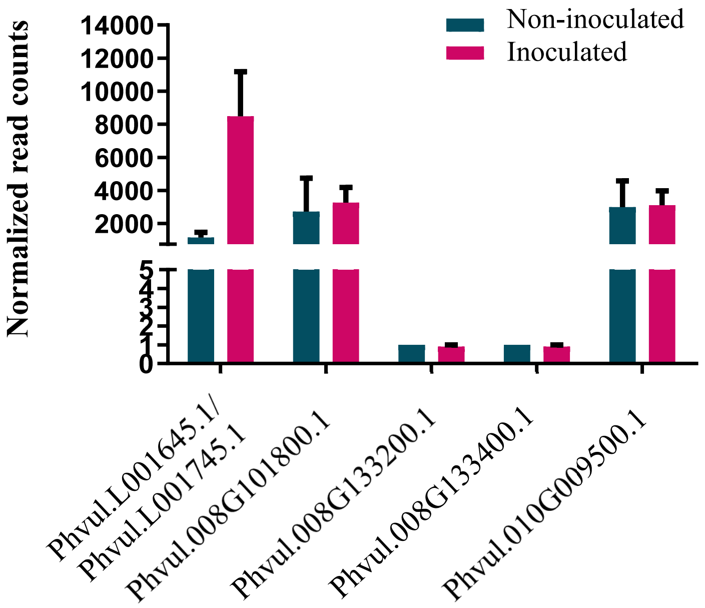

(B)

RPKM values

Experiment	Phvul.L001645.1.p/ Phvul.L001745.1.p	Phvul.010G009500.1	Phvul.008G101800.1	Phvul.008G133400.1	Phvul.008G133200.1
PvFY	18	7232	2004	64	60
PvYL	5	11313	730	0	0
PvL5	17	3166	284	0	0
PvN5	403	459	710	0	0
PvNE	331	3749	229	0	0
PvNI	1609	2142	164	0	0
PvRF	8242	1834	708	0	0
PvR5	6141	3708	671	1	1
PvRE	4481	2031	504	18	17
PvRI	5579	5138	567	0	0
PvRT	51	25	91	0	0
PvYR	2519	1450	301	0	0
PvS1	16	2039	1593	465	453
PvS2	4	3028	815	734	713

Leaves
Nodules
Roots
Seeds

High
Expression profile at PVGA
Low

Figure S5. Expression profiles of *in silico* transcripts of *PvMT* genes. (A) Normalized read counts of the six *PvMT* genes retrieved from our previous transcriptomic analysis of *P. vulgaris* roots inoculated with *R. tropici* (7 dpi) and non-inoculated (Fonseca-García et al., 2019). **(B)** Heatmap of the *PvMT* genes expression profiles in different organs and tissues of *P. vulgaris*. Expression was analyzed using the *Phaseolus vulgaris* Gene Expression Atlas (*Pv*GEA): PvFY, young flowers collected prior to floral emergence; PvYL, fully expanded 2nd trifoliate leaf tissue from plants provided with fertilizer; PvL5, leaf tissue collected 5 days after plants were inoculated with effective rhizobium; PvN5, pre-fixing (effective) nodules collected 5 days after inoculation; PvNE, Effectively fixing nodules collected 21 days after inoculation; PvNI, ineffectively fixing nodules collected 21 days after inoculation; PvR5, whole roots separated from 5-day-old pre-fixing nodules; PvRE, whole roots separated from fix+ nodules collected 21 days after inoculation; PvRI, whole roots separated from fix- nodules collected 21 days after inoculation; PvRT, root tips, 0.5 cm of tissue collected from fertilized plants at the 2nd trifoliate stage of development; PvYR, whole roots, including root tips, collected at the 2nd trifoliate stage of development; PvS1, stage 1 seeds between 6 and 7 mm across and approximately 50 mg: PvS2, stage 2 seeds between 8 and 10 mm across and between 140 and 150 mg.

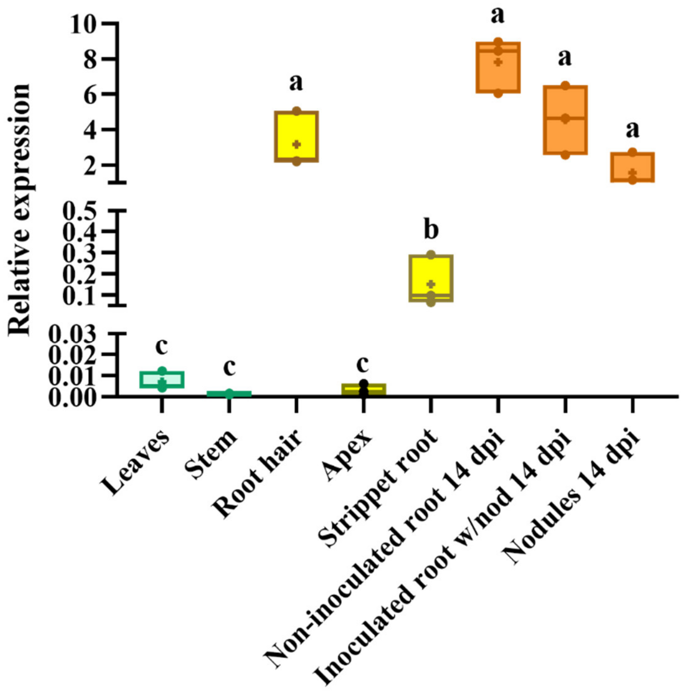

Figure S6. Expression profile of *PvMT1A* transcript in different *P. vulgaris* tissues. The expression of *PvMT1A* was evaluated in root hairs, apices, and stripped roots (roots remaining after the separation of root hairs) of seedlings harvested at 2 days post-germination; leaves, stems, and roots of non-inoculated plants at 14 days; and inoculated roots without nodules and nodules at 14 dpi. The top and bottom edges of the boxes delineate the first to third quartile, the horizontal line within the box represents the median, and the whiskers represent the smallest and largest outlier in the data set. The box plots represent three biological replicas (n = 120). Colors represent the plant section or the treatment of the samples: green, aboveground samples; yellow, belowground samples harvested at 2 days post-germination; orange, belowground samples harvested at 14 dpi non-inoculated or inoculated with *R. tropici*. The elongation factor *EF1α* gene was used as the endogenous reference gene. Different lowercase letters represent significant differences after Kruskal-Wallis test and Dunn's multiple comparison test ($P \leq 0.05$). Same letter represents there is no significant differences between the samples such as leaves, stem, and apex that have the letter c.

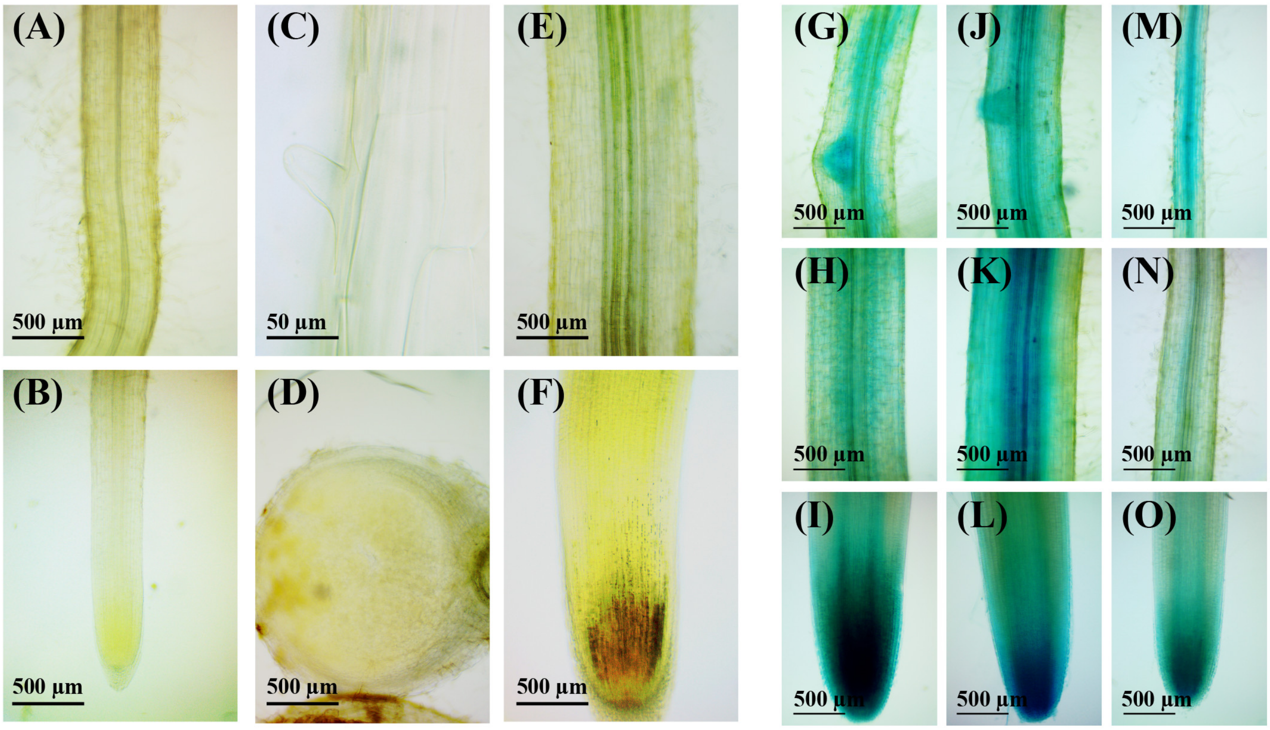

Figure S7. **Promoter activity analysis of *PvMT1A*. (A-F)** GUS activity in non-inoculated and inoculated hairy roots carrying the empty vector . **(A)** Main root and **(B)** its apex, at seven days non-inoculated. **(C)** Root hair at 7 dpi, **(D)** mature nodule at 30 dpi, **(E)** main root and **(F)** its apex at 30 dpi. **(G-I)** GUS activity in hairy roots carrying p*PvMT1A*::GUS:GFP construct. **(G)** Lateral root primordia of first order at seven days non-inoculated, **(H)** main root and **(I)** its apex at seven days non-inoculated. **(J)** Lateral root primordia of first order at 7 dpi, **(K)** main root and **(L)** its apex at 7 dpi. **(M)** Lateral root of of second order at 30 dpi, **(N)** lateral root of first order and **(O)** its apex at 30 dpi.

(A)

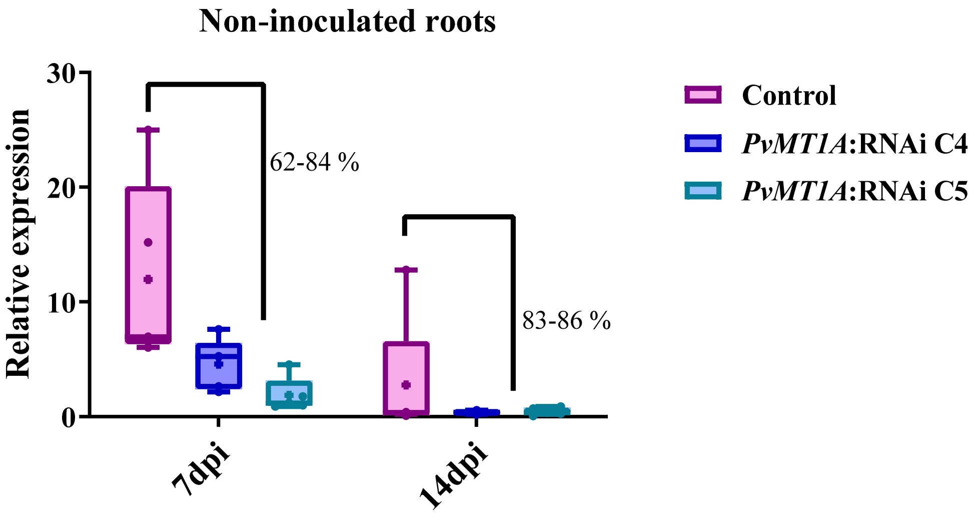

(B)

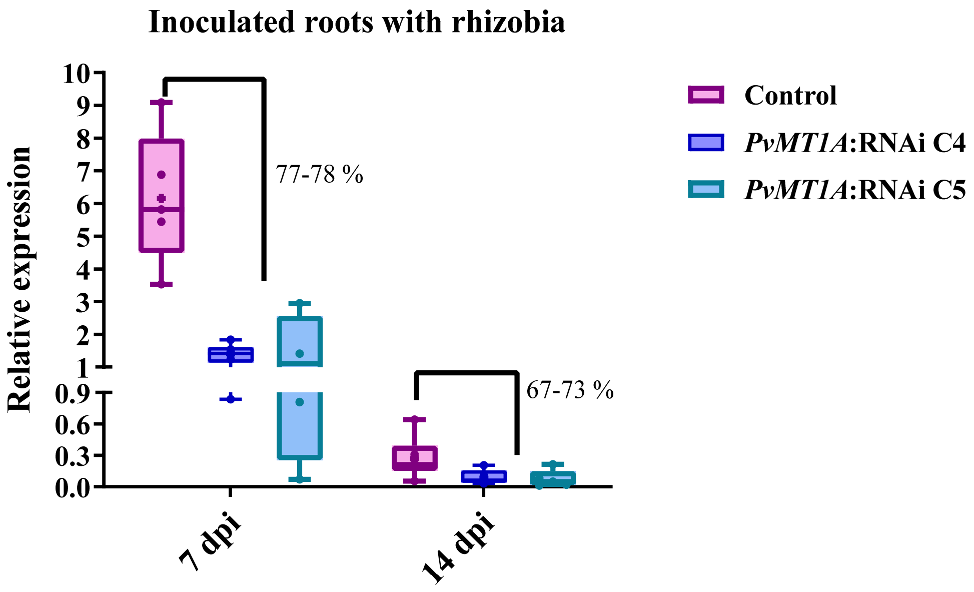

Figure S8. Validation of the gene silencing level in *PvMT1A*:RNAi transgenic roots. The transcripts abundance was analyzed by qPCR in transgenic roots transformed with the empty vector or with the *PvMT1A*:RNAi construct of **(A)** non-inoculated and **(B)** inoculated roots with *R. tropici* at 7 and 14 dpi. The elongation factor *EF1α* gene was used as an endogenous reference gene to normalize expression levels. The top and bottom edges of the boxes delineate the first to third quartile, the horizontal line within the box represents the median,

and the whiskers represent the smallest and largest outlier in the data set. The box plots represent two biological replicas (n = 6).

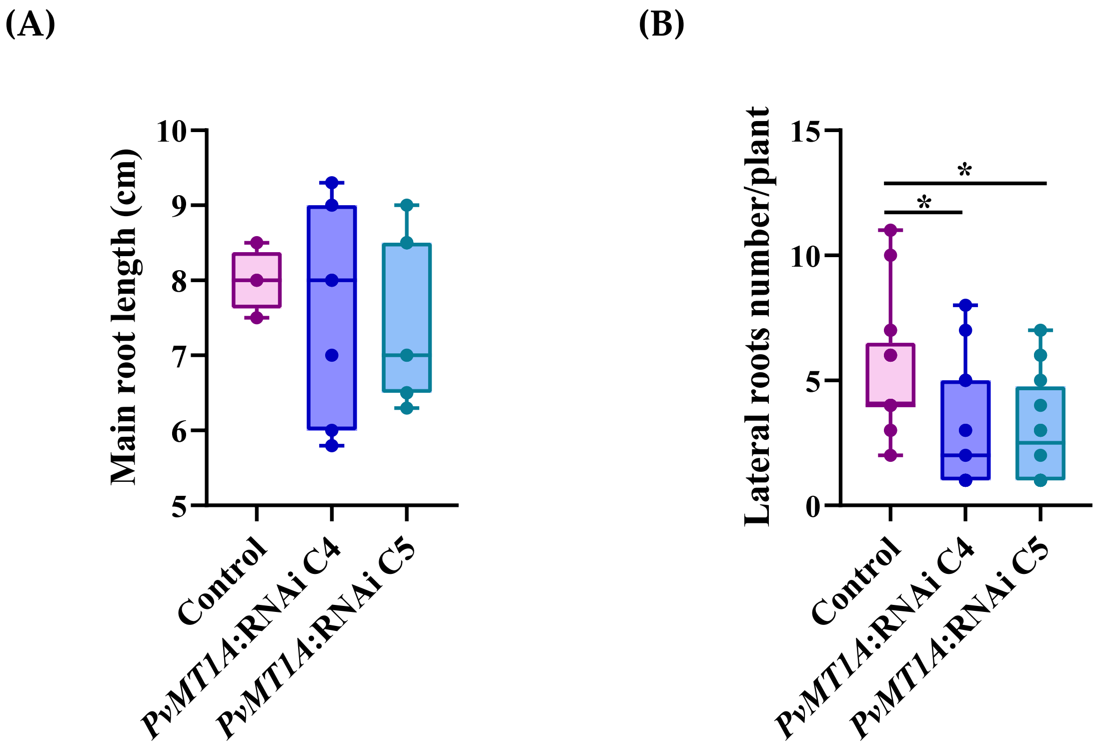

Figure S9. Effect of the downregulation of *PvMT1A* on the root development. (A) Main root length and **(B)** number of lateral roots per plant of composite plants carrying the empty vector or the *PvMT1A*:RNAi construct (clones C4 and C5) at 7 days in pots. The asterisks in the upper part represent significant differences after Kruskal-Wallis test and Dunn's multiple comparison test ($P \leq 0.05$).

(A) ***PvENOD2*** **(B)** ***PvENOD40***

Non-inoculated **Inoculated**

Figure S10. Expression profile analysis of early nodulin genes. (A) *PvENOD2* and **(B)** *PvENOD40* transcript levels were determined by qPCR of total RNA isolated from control and *PvMT1A*:RNAi transgenic roots non-inoculated and inoculated with *R. tropici* at 7 dpi. The elongation factor *EF1α* gene was used as an endogenous reference gene to normalize expression levels. The top and bottom edges of the boxes delineate the first to third quartile, the horizontal line within the box represents the median, and the whiskers represent the smallest and largest outlier in the data set. The box plots represent three biological replicas (n = 9). The asterisks at the top represent significant differences after the Mann Whitney test *$p \leq 0.05$, **$p \leq 0.01$, and ns = no significant difference.

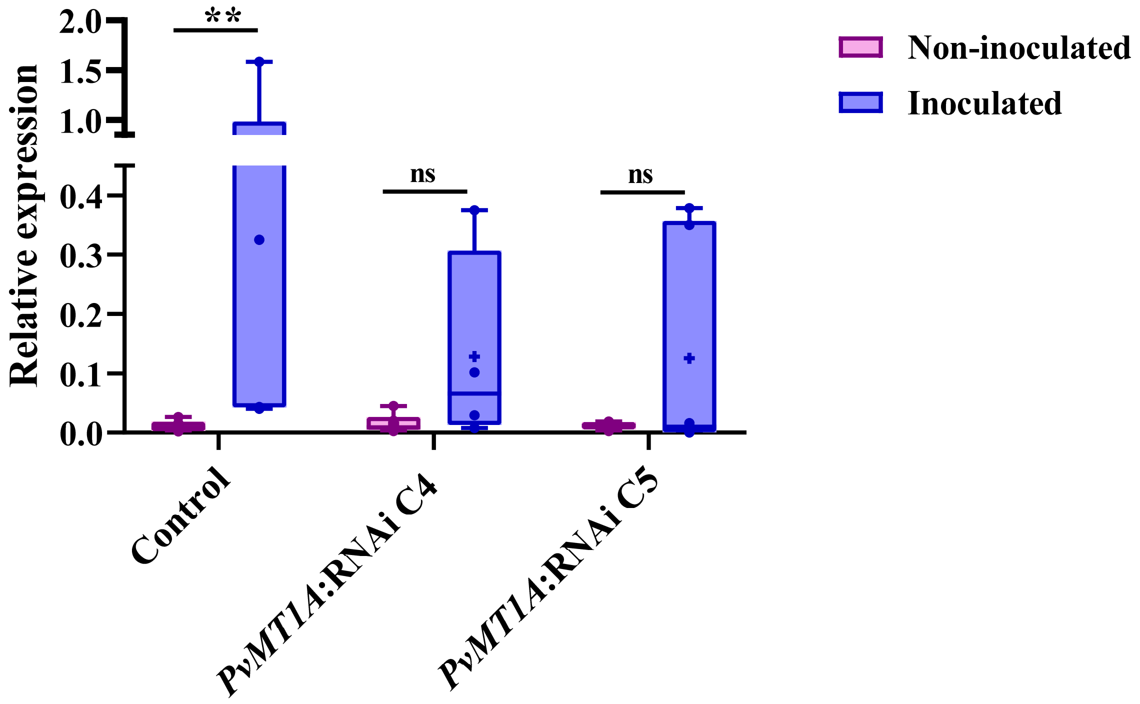

Figure S11. Expression profile analysis of the leghemoglobin gene. Leghemoglobin gene transcript levels were determined by qPCR of total RNA isolated from control and *PvMT1A*:RNAi transgenic roots non-inoculated and inoculated with *R. tropici* at 14 dpi. The elongation factor gene *EF1α* was used as an endogenous reference gene to normalize expression levels. The top and bottom edges of the boxes delineate the first to third quartile, the horizontal line within the box represents the median, and the whiskers represent the smallest and largest outlier in the data set. The box plots represent two biological replicates (n = 6). The asterisks at the top represent significant differences after the Mann Whitney test *$p \leq 0.05$ and ns = no significant difference.

(A) ***PvRbohA*** **(B)** ***PvRbohB***

Non-inoculated **Inoculated**

Figure S12. Expression profile of transcripts of the *PvRboh* genes. The transcript levels of the **(A)** *PvRbohA* and **(B)** *PvRbohB* genes were determined by qPCR of total RNA isolated from control and *PvMT1A*:RNAi transgenic roots non-inoculated and inoculated with *R. tropici* at 7 dpi. The elongation factor *EF1α* gene was used as an endogenous reference gene to normalize expression levels. The top and bottom edges of the boxes delineate the first to third quartile, the horizontal line within the box represents the median, and the whiskers represent the smallest and largest outlier in the data set. The box plots represent three biological replicas (n = 9). The asterisks at the top represent significant differences after the Mann Whitney test $*p \leq 0.05$ and ns = no significant difference.

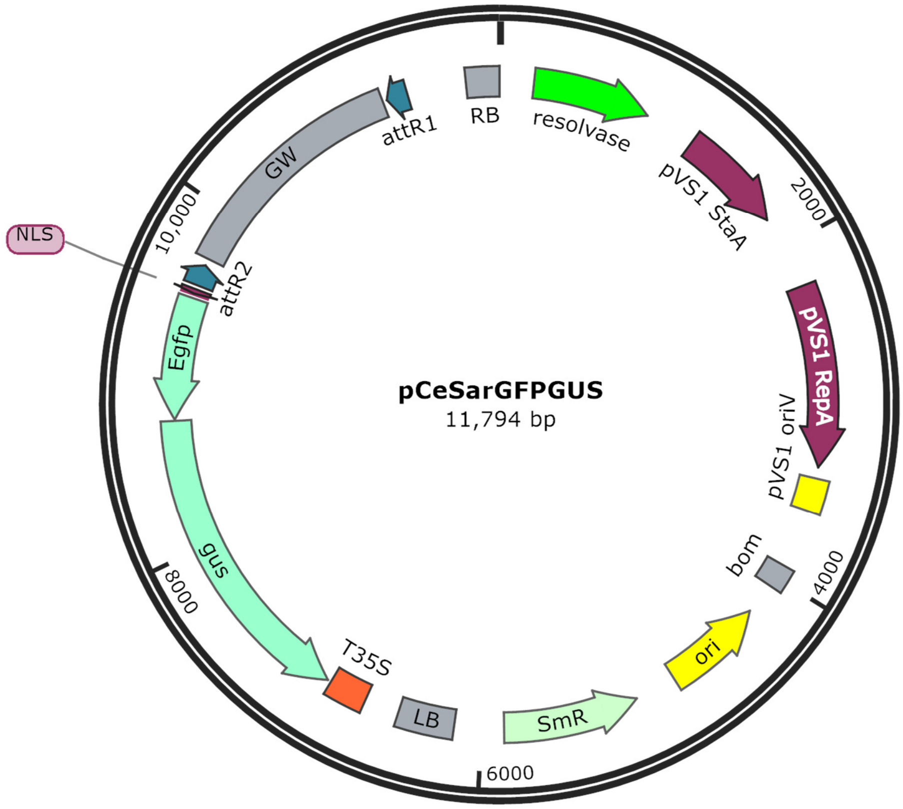

Figure S13. *In silico* map of the pCeSar vector. This plasmid was derived from the pBGWSF7.0 vector (Karimi et al., 2002); a nuclear localization signal was inserted before the EGFP sequence. The image was created with SnapGene version 5.2.4 software.

References

1. Fonseca-García, C.; Zayas, A.E.; Montiel, J.; Nava, N.; Sánchez, F.; Quinto, C. Transcriptome analysis of the differential effect of the NADPH oxidase gene RbohB in Phaseolus vulgaris roots following Rhizobium tropici and Rhizophagus irregularis inoculation. *BMC Genom.* **2019**, *20*, 1–18. https://doi.org/10.1186/s12864-019-6162-7

2. Karimi, M.; Inzé, D.; Depicker, A. GATEWAY vectors for Agrobacterium-mediated plant transformation. *Trends Plant Sci.* **2002**, *7*, 193–195.
